# My virtual home: needs of patients in palliative cancer care and content effects of individualized virtual reality – a mixed methods study protocol

**DOI:** 10.1186/s12904-023-01297-z

**Published:** 2023-10-31

**Authors:** Christina Gerlach, Anja Greinacher, Bernd Alt-Epping, Cornelia Wrzus

**Affiliations:** 1 Department of Palliative Medicine, Heidelberg University Hospital, Im Neuenheimer Feld 305, 69120 Heidelberg, Germany; 2 Institute of Medical Psychology, Heidelberg University Hospital, Bergheimer Straße 20, 69115 Heidelberg, Germany; 3 Psychological Institute and Network Aging Research, Ruprecht Karls University of Heidelberg, Bergheimer Str. 20, 69115 Heidelberg, Germany

**Keywords:** Virtual reality, Palliative care, Oncology, Personalized therapy

## Abstract

**Background:**

The desire to be at home is one of the most important needs of patients with advanced, incurable cancers. However, palliative cancer patients may require inpatient hospital care for medical reasons. Virtual reality (VR) could provide an approximation to the individuals’ home environment.

**Methods:**

The project consists of 3 parts. All parts are supported by the patient advisory board. In the 1st part of the project, we interview patients, relatives, and the patient advisory board about their wishes and concerns regarding the project. In the 2nd part of the project, patients are offered to view 360° VR videos of their choice (their home, relatives, others if applicable). Effects and side-effects of the intervention are assessed with validated instruments (MIDOS, MDBF, SSQ, SPES). Diagnosis, treatment adherence, medication, and vegetative functionality is determined from the medical records of the patients. In the 3rd part of the project, the results of the study will be discussed with patients, relatives, health care professionals and the patient advisory board regarding implementation.

**Discussion:**

This study is the first to investigate whether individualized VR videos have additional benefits over generic VR nature videos on symptom relief, well-being, treatment satisfaction, and adherence in patients with palliative cancer care. A strength of the study is that we can incorporate the results of each part of the project into the subsequent project parts. However, the generalizability might be limited as this is a single-centred study.

**Trial registration:**

Registered at German Clinical Trials Register (Deutsches Register Klinischer Studien; DRKS); registration number: DRKS00032172; registration date: 11/07/2023 https://drks.de/search/de/trial/DRKS00032172.

**Supplementary Information:**

The online version contains supplementary material available at 10.1186/s12904-023-01297-z.

## Background

To be at home is one of the most important desires of patients with advanced, incurable cancer [1, 2]. Therefore, spending as much time as possible at home is gaining importance in palliative cancer care [3, 4]. Patients express a desire for moments of normalcy in which they are allowed to be a person and do not feel reduced to their patient role [5]. Such normalcy may be facilitated by the familiar home environment.

Still, palliative cancer patients may need inpatient hospital care for medical reasons. In this case, virtual reality (VR) could provide a bridge to the individual home environment, when patients view their home in VR and ideally “feel at home”. In VR, immersion refers to the feeling of being in a different world [6]. In the intended application of our study, this world is the places the patients’ desire. VR applications distract from visual and auditory stimuli that can cause anxiety, longing, and other negative feelings, and have a positive effect on symptom relief and well-being [7–11]. For example, patients undergoing chemotherapy reported reduction of anxiety due to altered perception of time, as well as feelings of fun and enjoyment [12, 13]. Cancer patients who view nature images via VR headsets experience less pain and a higher sense of well-being [14]. Here, the nature images are identical for all individuals. Initial research on the potential of personalized VR in palliative care did not show significant results, which was attributed to the limited selection of VR video scenarios [15]. The use of home VR videos could increase the effect of individual images [16, 17], and respond to the desire to feel at home.

The aim of the study is to assess if VR videos with content tailored to the individual palliative cancer inpatient’s desire to be at home are accepted and suitable to improve their quality of life. The specific objectives of the study are:

- Exploration of patient and family views about desired content and concerns of a VR intervention, and consultation with the patient advisory committee;
- Clinical testing of an individualized VR intervention for palliative cancer patients;
- Patient-and-Public-Involvement (PPI) with patients, families, patient advisory board, and the involved hospital wards for a reciprocal exchange of findings, which will be used for the further implementation.

## Methods

This study is composed of three successive parts that build upon each other, continuously accompanied by the patient advisory board, which is already involved in the writing of the study protocol. Both, qualitative and quantitative methods will be applied.

### Procedure

All eligible persons for the three parts of the project will be informed about the study in general and the particular project part. After informed consent the participants will be interviewed (I, III), or experience the VR videos (II) with outcome assessments before and after the intervention. Results will be discussed with the patient advisory board after each of the project parts to inform the following project part.

### Project part I: PPI on desired content and concerns of a VR intervention

Aim, study design and setting: We conduct semi-structured interviews with inpatients and family members to explore wishes and concerns regarding VR; further, we ask specifically about 360° video recordings of one’s own home and family. The setting are units of the Heidelberg University Hospital that care for palliative cancer patients. In a consultative session with members of the patient advisory board, the contents of the interview will be discussed.

Participants: Palliative cancer patients at Heidelberg University Hospital and their family members are invited to the interview. They are identified by the attending physician of the unit (palliative care, radiotherapy, haematology). Inclusion criteria are age ≥ 18 years; patients or family members of patients diagnosed with an oncological or haematological disease that is not curable or is associated with an uncertain prognosis; capacity to consent. Exclusion criteria for patients include patients, who are in a poor general condition or too stressed to participate in any study as assessed by the health care professionals in charge; cognitive or communication deficits; life expectancy of only a few days. Exclusion criteria for family members include relatives who feel too burdened to participate in a study; cognitive or communication deficits; and being a family member of a dying patient.

Interviews: The semi-structured interview guide includes questions about wishes, concerns, and possible benefits of personalized VR videos in palliative cancer care. To respond to possible emotional distress, further topics and thoughts that complement from the study questions are given space and respect. The informed consent as well as the interview will take approximately 15–20 min. Information regarding the medical condition of participating patients is pseudonymized and transferred to a digital file by persons bound to confidentiality on the basis of the patient file. Patients will be asked for demographic information and length of stay (recorded pseudonymously).

Data analysis: We conduct interviews until there is data saturation in terms of no new information related to the research question [18]. To achieve this, we will continuously reflect in our interdisciplinary working group on the interviewees’ responses throughout the interview phase. We estimate the number of 20 interviews based on previous experiences with these participant groups [19]. The interviews will be audio-recorded, transcribed and analysed (content analysis). The focus points of the interview are set deductively, i.e. theory-guided, and form the framework for the category system in the analysis. The subcategories are developed inductively, i.e. from the interview material [20]. We apply descriptive quantitative methods (frequency distribution, mean, median, interquartile range) to evaluate the sociodemographic variables and data on the medical condition for the description of the purposive sample.

### Project part II: assessment of the experience with the individualized VR-intervention

Aim, study design and setting: In a prospective clinical cohort study, we assess the influence of personalized VR videos on symptoms, well-being, and vegetative functionality. Data will be collected using questionnaires and patient record analysis. First and foremost, we want to ensure patient safety. Thus, the aim is to prove a non-inferiority of the intervention in this vulnerable group and to start with 10 patients at least. Further, we want to observe whether ceiling effects occur in terms of well-being in this patient group, and to match the subjective verbal judgment of the patients regarding well-being after the VR-experience compared to baseline to reflect on the assumption that the minimally clinically important difference for well-being as assessed with the MDBF (Multi Dimensional Well-being Questionnaire) [21] is a 0.5 standard deviation or actually more prior to the sample size calculation for a power of 0.8 with a one sided significance level of 0.05, resp. effect size in a consecutive larger sample [22, 23]. This piloting is important because from our assumption so far the sample size would range between 30 and 130 participants [24, 25].

Participants: The same inclusion and exclusion criteria apply as in project part I.

Intervention: We create personalized VR videos as requested from the patients, probably from the patient’s home or family members. After the videos are created, the patients watch the videos using VR headsets while being cared for in a hospital unit. The videos are edited to a length of about 3–15 min. The patients watch the generic nature video for comparison before or after the personalized video (1:1 randomization). Before and after watching the videos, the patients answer the questionnaires using a tablet computer; support by a trained research assistant is provided to the comfort of the patients. The whole procedure of VR application takes about 20–30 min. If desired, patients can watch the personalized video repeatedly on different days for a maximum duration of 14 days. If patients are interested, they will be provided with the video file.

Questionnaires: Symptom perception (minimal documentation system, MIDOS) [26], well-being (MDBF Multidimensional Well-Being Questionnaire) [21], subjective benefit of VR videos (2 self-developed items), cybersickness (SSQ Simulator Sickness Questionnaire) [27], presence experience (SPES Spatial Presence Experience Scale) [28].

In addition to interviewing patients after viewing VR videos, information on disease, treatment adherence, pain medication, and vegetative functionality will be collected from patient records to avoid further, potentially burdensome physical examinations during study participation.

Data analysis: The analysis is performed with the total data set reflecting the intention-to-treat principle. Descriptive methods are used for the analysis of all collected data (frequency distributions, mean, median, interquartile). For the pre/post comparisons of the outcomes (well-being, symptom perception, treatment experience, treatment adherence, pain medication, autonomic functionality), with well-being as the primary outcome, non-parametric Wilcoxon signed-rank t-tests for repeated measures data are calculated. Analysis is performed using IBM SPSS Statistics and R (r-project.org).

### Project part III: PPI to inform VR implementation in palliative cancer care

Aim, study design and setting: PPI will be used for a reciprocal exchange of findings, and the further implementation of the individualized approach for VR in palliative cancer care.

Participants and procedure: Patients, patients’ family members, the patient advisory board, and healthcare professionals from the participating hospital departments are invited to exchange views on strengths, weaknesses, opportunities and threats (SWOT) in the context of implementation of the personalized VR intervention based on the results from the project part II.

Data analysis: We structure the results from the discussion by a SWOT analysis (strength, weaknesses, opportunities, threats) to inform an implementation strategy.

### Data management and dissemination

#### Confidentiality

The names of participants and all other confidential information are subject to medical confidentiality and the provisions of the Data Protection Regulation (Datenschutzgrundverordnung DSGVO) and the State or Federal Data Protection Act (LDSG or BDSG). Data collected during the study will be kept anonymously in accordance with good scientific practice for 10 years after the end of the study (until 2034). The data will be used exclusively for the purposes of this study. All data will be stored pseudonymously in access-restricted drives on secured computers and servers of the University of Heidelberg and the University Hospital, Department of Palliative Care, of the scientists involved in the project. The pseudonymization keys as well as signed consent forms are stored in the locked steel cabinet of a project partner (CW). A digital file of the pseudonymization key, which contains only ID and name of the participating persons, is double password protected and stored in the secure sync-and share service for collaboration within the university, hosted locally in the cloud by the university data center. Only the scientists involved in the project have access to the pseudonymization keys.

Participant data may only be passed on in pseudonymized form, which is anonymized by destroying the pseudonymization list three months after the end of the data collection. Third parties are not given access to original documents.

#### Dissemination policy

Study results will be published in peer-reviewed, indexed, journals using an open access format, and the results will be presented at national and international conferences. Authorship eligibility will be in accordance with The International Committee of Medical Journal Editors. The National Center for Tumor Diseases Heidelberg foundation supports this study and we will work with them and the Patient Advisory Board to disseminate findings easily accessible for patients and healthcare professionals.

## Discussion

This study aims to investigate whether individual VR videos provide benefits in terms of symptom relief, well-being, treatment satisfaction, and treatment adherence in palliative cancer care. The novelty of this project are the individualized VR videos as in previous research with palliative patients only generic videos, such as nature shots, were used.

A further strength of the study is that the patients’ opinions as well as those of the relatives and the patient advisory board are considered, i.e., heavily involving those concerned by the intervention in its development. The combination of qualitative and quantitative method allows to inform the subsequent project parts: In the first part, we start with qualitative interviews to gain an in-depth understanding of the topic. We ask not only about wishes and needs, but also about worries and concerns. The results of the first part enable us to respond with a high degree of flexibility to the needs of the patients in the second part of the study. Moreover, we ensure patient safety when we assess and compare cybersickness, symptoms, and use of medication for symptoms before and after the intervention with no tolerance for a decline of the patient’s subjective wellbeing. The study is designed to implement VR video use tailored to the needs of patients feasible not only under laboratory conditions but in everyday clinical practice.

However, the generalisability of our study results will be limited because we perform a single centre study. Nevertheless, the experiences from the project may inform others who want to adapt the intervention to their site or to other palliative care samples. In terms of the sample, we suspect that particularly highly burdened patients will not participate in the study, although they would like to use the VR intervention outside of a trial. For this reason, we involve relatives and the patient representatives’ experiences and attitudes as a proxy. Whether older patients will be more likely to decline study participation because of restraints towards new technology is an apprehension that could not be clarified in former studies applying VR interventions to older people [29], and we aim to consider the particular needs of vulnerable and old people to operate the VR intervention.

The NCT funding board reviewed a summary of this study to ensure its aims and objectives, study design and outcome are suited and promising to improve the care of cancer patients. The Institute for Biometrics of the Heidelberg University Hospital reviewed the quantitative data analysis plan and recommended descriptive methods. The anthropologist and research coordinator of the Department of Palliative Care of the Heidelberg University Hospital reviewed the protocol regarding the qualitative methodology and mixed methods approach. Further, three members of the NCT patient advisory board informed the interview guide, the selection of questionnaires, and the sheets for informed consent. They found it important to explore the potential of individualized VR-videos to alleviate the situation of palliative inpatients with cancer. In this context, they welcomed the approach to find out the wishes and concerns of patients and relatives related to the intervention to guide its implementation.

### Electronic supplementary material

Below is the link to the electronic supplementary material.

Supplementary Material 1

## Data Availability

Model participant information sheet and consent form, interview guides, and examples from the input window for the outcome assessments can be provided on request from the corresponding author.

## List of abbreviations

PPI: Patient and-Public-Involvement
VR: Virtual Reality

## Declarations

### Protocol version

2.0 27/04/2023.

## References

[1] Gomes B, Calanzani N, Gysels M, Hall S, Higginson IJ (2013). Heterogeneity and changes in preferences for dying at home: a systematic review. BMC Palliat care.

[2] Townsend J, Frank A, Fermont D, Dyer S, Karran O, Walgrove A, Piper M (1990). Terminal cancer care and patients’ preference for place of death: a prospective study. BMJ.

[3] Alt-Epping B, Nauck F (2012). Der Wunsch Des Patienten–Ein eigenständiger Normativer Faktor in Der Klinischen Therapieentscheidung?. Ethik in Der Medizin.

[4] Yun YH, Kim K-N, Sim J-A, Kang E, Lee J, Choo J, Yoo SH, Kim M, Kim YA, Kang BD (2019). Correction to: priorities of a good death according to cancer patients, their family caregivers, physicians, and the general population: a nationwide survey. Support Care Cancer.

[5] Chochinov HM, McClement S, Hack T, Thompson G, Dufault B, Harlos M (2015). Eliciting personhood within clinical practice: effects on patients, families, and health care providers. J Pain Symptom Manag.

[6] Slater M, Wilbur S (1997). A framework for immersive virtual environments (FIVE): speculations on the role of presence in virtual environments. Presence: Teleoperators & Virtual Environments.

[7] Atzori B, Hoffman HG, Vagnoli L, Patterson DR, Alhalabi W, Messeri A, Lauro Grotto R (2018). Virtual reality analgesia during venipuncture in pediatric patients with onco-hematological Diseases. Front Psychol.

[8] Hoffmann S. Somatisierungsstörung und somatoforme Störungen - Herkunft der Konzepte und ihre Abbildung in den neuen diagnostischen Glossaren. In: *Somatoforme Störungen: Theoretisches Verständnis und therapeutische Praxis* edn. Edited by Rudolf G, Henningsen P. Stuttgart: Schattauer; 1998: 3–12.

[9] Jeffs D, Dorman D, Brown S, Files A, Graves T, Kirk E, Meredith-Neve S, Sanders J, White B, Swearingen CJ (2014). Effect of virtual reality on adolescent pain during burn wound care. J Burn Care Res.

[10] Ridout B, Kelson J, Campbell A, Steinbeck K (2021). Effectiveness of virtual reality interventions for adolescent patients in hospital settings: systematic review. J Med Internet Res.

[11] Soltani M, Drever SA, Hoffman HG, Sharar SR, Wiechman SA, Jensen MP, Patterson DR (2018). Virtual reality analgesia for burn joint flexibility: a randomized controlled trial. Rehabil Psychol.

[12] Schneider SM, Hood LE. Virtual reality: a distraction intervention for chemotherapy. Oncology nursing forum: 2007: NIH Public Access; 2007: 39. 10.1188/07.ONF.39-46 PMC2121303 17562631

[13] Schneider SM, Prince-Paul M, Allen MJ, Silverman P, Talaba D. Virtual reality as a distraction intervention for women receiving chemotherapy. In: *Oncology nursing forum: 2004*; 2004. 10.1188/04.ONF.81-88 14722591

[14] Pittara M, Matsangidou M, Stylianides K, Petkov N, Pattichis CS (2020). Virtual reality for pain management in cancer: a comprehensive review. IEEE Access.

[15] Perna M, Letizia MSW, Lund S, White N, Minton O (2021). The potential of personalized virtual reality in palliative care: a feasibility trial. Am J Hospice Palliat Medicine®.

[16] Fedorovskaya EA, Miller P, Prabhu G, Horwitz C, Matraszek T, Parks P, Blazey R, Endrikhovski S. Affective imaging: psychological and physiological reactions to individually chosen images. Human vision and electronic imaging VI: 2001: SPIE; 2001: 524–32.

[17] Sekhavat YA, Nomani P. A comparison of active and Passive virtual reality exposure scenarios to elicit social anxiety. Int J Serious Games 2017, 4(2).

[18] Braun V, Clarke V (2021). To saturate or not to saturate? Questioning data saturation as a useful concept for thematic analysis and sample-size rationales. Qualitative Res Sport Exerc Health.

[19] Gerlach C, Ullrich A, Berges N, Bausewein C, Oechsle K, Hodiamont F, for the PallPanStudy Group. The impact of the SARS-CoV-2 pandemic on the needs of non-infected patients and their families in palliative care—interviews with those concerned. J Clin Med. 2022;11(13):3863. 10.3390/jcm11133863 PMC9267922 35807148

[20] Mayring P. Qualitative inhaltsanalyse. Handbuch qualitative Forschung in Der Psychologie. edn.: Springer; 2010: 601–13.

[21] Steyer R, Schwenkmezger P, Notz P, Eid M. Testtheoretische Analysen des Mehrdimensionalen Befindlichkeitsfragebogen (MDBF). *Diagnostica* 1994.

[22] Norman GR, Sloan JA, Wyrwich KW (2003). Interpretation of changes in Health-related quality of life: the remarkable universality of half a standard deviation. Med Care.

[23] de Vet HC, Terwee CB, Ostelo RW, Beckerman H, Knol DL, Bouter LM (2006). Minimal changes in health status questionnaires: distinction between minimally detectable change and minimally important change. Health Qual Life Outcomes.

[24] Maxwell SE, Kelley K, Rausch JR (2008). Sample size planning for Statistical Power and accuracy in parameter estimation. Ann Rev Psychol.

[25] Altman DG (1999). Appendix III clinical trials, sample size. Practical Statistics for Medical Research.

[26] Stiel S, Matthes M, Bertram L, Ostgathe C, Elsner F, Radbruch L (2010). Validation of the new version of the minimal documentation system (MIDOS) for patients in palliative care: the German version of the Edmonton symptom assessment scale (ESAS). Der Schmerz.

[27] Kennedy RS, Lane NE, Berbaum KS, Lilienthal MG (1993). Simulator sickness questionnaire: an enhanced method for quantifying simulator sickness. Int J Aviat Psychol.

[28] Hartmann T, Wirth W, Schramm H, Klimmt C, Vorderer P, Gysbers A, Böcking S, Ravaja N, Laarni J, Saari T (2016). The spatial presence experience scale (SPES): a short self-report measure for diverse media settings. J Media Psychol.

[29] Neyer FJ, Felber J, Gebhardt C (2012). Development and validation of a brief measure of technology commitment. Diagnostica.

[30] World Medical Association. Declaration of Helsinki: ethical principles for medical research involving human subjects. 64th WMA General Assembly, Fortaleza, Brazil. WMA [Web Site] Outubro; 2013.

